# Reducing Alert Fatigue by Sharing Low-Level Alerts With Patients and Enhancing Collaborative Decision Making Using Blockchain Technology: Scoping Review and Proposed Framework (MedAlert)

**DOI:** 10.2196/22013

**Published:** 2020-10-28

**Authors:** Paul Kengfai Wan, Abylay Satybaldy, Lizhen Huang, Halvor Holtskog, Mariusz Nowostawski

**Affiliations:** 1 Department of Manufacturing and Civil Engineering Norwegian University of Science and Technology Gjøvik Norway; 2 Department of Computer Science Norwegian University of Science and Technology Gjøvik Norway; 3 Department of Industrial Economics and Technology Management Norwegian University of Science and Technology Gjøvik Norway

**Keywords:** blockchain, health care, alert fatigue, clinical decision support, smart contracts, information sharing

## Abstract

**Background:**

Clinical decision support (CDS) is a tool that helps clinicians in decision making by generating clinical alerts to supplement their previous knowledge and experience. However, CDS generates a high volume of irrelevant alerts, resulting in alert fatigue among clinicians. Alert fatigue is the mental state of alerts consuming too much time and mental energy, which often results in relevant alerts being overridden unjustifiably, along with clinically irrelevant ones. Consequently, clinicians become less responsive to important alerts, which opens the door to medication errors.

**Objective:**

This study aims to explore how a blockchain-based solution can reduce alert fatigue through collaborative alert sharing in the health sector, thus improving overall health care quality for both patients and clinicians.

**Methods:**

We have designed a 4-step approach to answer this research question. First, we identified five potential challenges based on the published literature through a scoping review. Second, a framework is designed to reduce alert fatigue by addressing the identified challenges with different digital components. Third, an evaluation is made by comparing MedAlert with other proposed solutions. Finally, the limitations and future work are also discussed.

**Results:**

Of the 341 academic papers collected, 8 were selected and analyzed. MedAlert securely distributes low-level (nonlife-threatening) clinical alerts to patients, enabling a collaborative clinical decision. Among the solutions in our framework, Hyperledger (private permissioned blockchain) and BankID (federated digital identity management) have been selected to overcome challenges such as data integrity, user identity, and privacy issues.

**Conclusions:**

MedAlert can reduce alert fatigue by attracting the attention of patients and clinicians, instead of solely reducing the total number of alerts. MedAlert offers other advantages, such as ensuring a higher degree of patient privacy and faster transaction times compared with other frameworks. This framework may not be suitable for elderly patients who are not technology savvy or in-patients. Future work in validating this framework based on real health care scenarios is needed to provide the performance evaluations of MedAlert and thus gain support for the better development of this idea.

## Introduction

### Background

Clinical decision support (CDS) is a tool to facilitate medical decision making by generating clinical alerts [1], ranging from simple medication-specific alerts based on stored clinical rules and information to more complex patient-specific alerts by integrating CDS with electronic health records (EHRs) [2]. For example, CDS warns clinicians by generating an alert if a new prescription poses a threat to patients [3]. This real-time alert disrupts the workflow and draws clinicians’ attention so they can evaluate and make appropriate decisions in a quick and efficient manner [4]. CDS has replaced previous situations in which clinicians make decisions solely on the basis of their knowledge and past experience [5]. CDS is now considered an essential health information technology that improves the overall quality of health care [6]. However, current CDS tools generate a high volume of irrelevant alerts, resulting in alert fatigue [7].

Alert fatigue or alert burden is defined as the mental state that results when alerts or reminders consume too much time and mental energy, which can cause clinicians to override or ignore both clinically irrelevant and relevant alerts unjustifiably [8]. Clinicians are now drowning with alerts and gradually becoming less responsive to and less respectful of them [9]. This is mainly because generated alerts are mostly irrelevant or low priority, and fortunately, they are not life threatening. In the long term, these *cry-wolf* alerts have desensitized clinicians, resulting in high overriding rates ranging between 77% and 90% [10-12], which opens the door to preventable medication errors.

Alert fatigue started becoming increasingly common in the health care sector decades ago and is now widely recognized as a national concern, often due to the lack of a corresponding action plan [13]. CDS failures and errors caused by individuals have resulted in direct costs of more than US $20 billion in the United States [14,15]. Alert fatigue is perceived as a major problem because it extends beyond the health care industry. Other sectors, such as off-shore oil drilling [16] and heating, ventilation, and air-conditioning systems in buildings [17], are also experiencing alert fatigue. For example, fault detection systems generate high volumes of alerts, leading to operator alert fatigue and resulting in energy wastage in buildings. Currently, there is a persistent upward trend and increasing requests for new alerts [13], which does not help alert fatigue. This only exacerbates the alert fatigue and makes it more widespread.

Overriding alerts is clinically appropriate if the alert generated is incorrect [7]. However, due to the low specificity and high volume of alerts generated by CDS, relevant alerts may also be dismissed, resulting in preventable prescription errors and adverse drug events. Deactivation [18] or running low-priority alerts in silence [19] are among the suggestions for reducing alert fatigue. However, these approaches in managing alerts effectively are difficult because of strict regulatory bodies and other external pressures. Many are in fact pushing for more rather than fewer alerts to reduce or avoid preventable medication errors [13].

In Norway, approximately 12% of patient harm is caused by the incorrect use of drugs [20]. One in three elderly people have been given the wrong medication, and an estimated one thousand deaths per year are thought to be due to medication errors, despite the use of e-prescriptions [21,22]. During a meeting at the Norwegian University of Science and Technology (NTNU), a health care representative from Innlandet Hospital presented in his presentation that approximately 8% of total health care spending went on correcting medication errors within the Innlandet region. [23]. We, therefore, agree with Wright et al [24] that the health care sector can only benefit from the potential value of CDS-generated alerts when they are well designed and properly implemented. Thus, there is a need to seek an alternative, innovative approach to improve the management of clinical alerts and reduce alert fatigue among clinicians.

### Objectives

Blockchain technology has gained attention as a potential solution in the health care sector, mainly due to its potential in moving toward collaborative treatments and decision making [25-27]. A large range of literature has been published anticipating this technology with a view to improving the health sector with respect to the overall well-being of clinicians and the quality of patients’ health care by sharing medical records and history [26]. However, studies focusing on clinical alerts using blockchain remain limited. This has led us to our main research question in this paper, which is to explore and understand how a blockchain-based solution can help to reduce alert fatigue in the health sector by sharing alerts and thus enhancing collaborative decision making. To answer this question, we designed a 4-step approach, which is explained in the *Methods* section.

## Methods

### Design Approach

**Figure 1:**
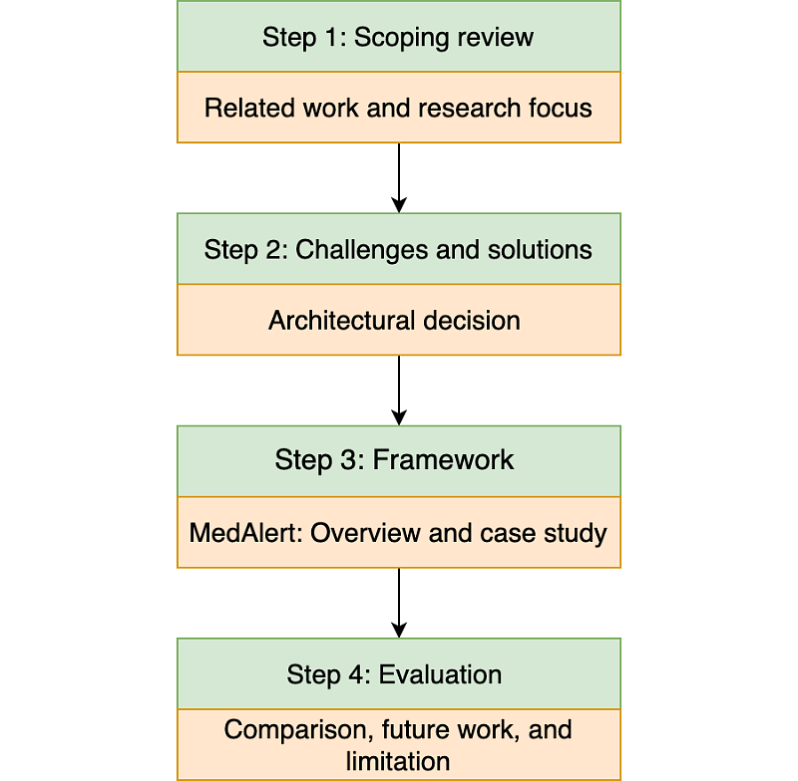
Research design process flow: 4-step approach.

The 4-step approach, shown in Figure 1, is designed to answer our research question and explain how the paper is organized. The first step is to conduct a scoping review to explore the current state of the art in this area. The literature we finally selected and the existing solutions we have chosen are then analyzed in step 1. Step 2 is designed to identify potential challenges and technical solutions for reducing alert fatigue. Architectural decisions are explained in this step. The framework is designed in step 3. An overview of MedAlert, together with a case study, is elaborated in this step. Finally, the framework is evaluated by comparing it with other proposed solutions. The comparison, future work, limitations, and benefits are also discussed in step 4.

### Scoping Review: Search Strategy

A scoping review was conducted with the aim of exploring the current state of the art in academic research with the widest possible coverage of all the published literature. The reporting of this scoping review was guided by PRISMA-ScR (Preferred Reporting Items for Systematic Reviews and Meta-analysis extension for Scoping Reviews) [28]. We performed searches on 2 bibliographic databases, Scopus and PubMed. To be as comprehensive as possible, generic keyword strings such as *blockchain*, *clinical decision support*, *alert burden*, and *alert fatigue* were used as search criteria. Multimedia Appendix 1 details the structures of the keyword strings.

**Figure 2:**
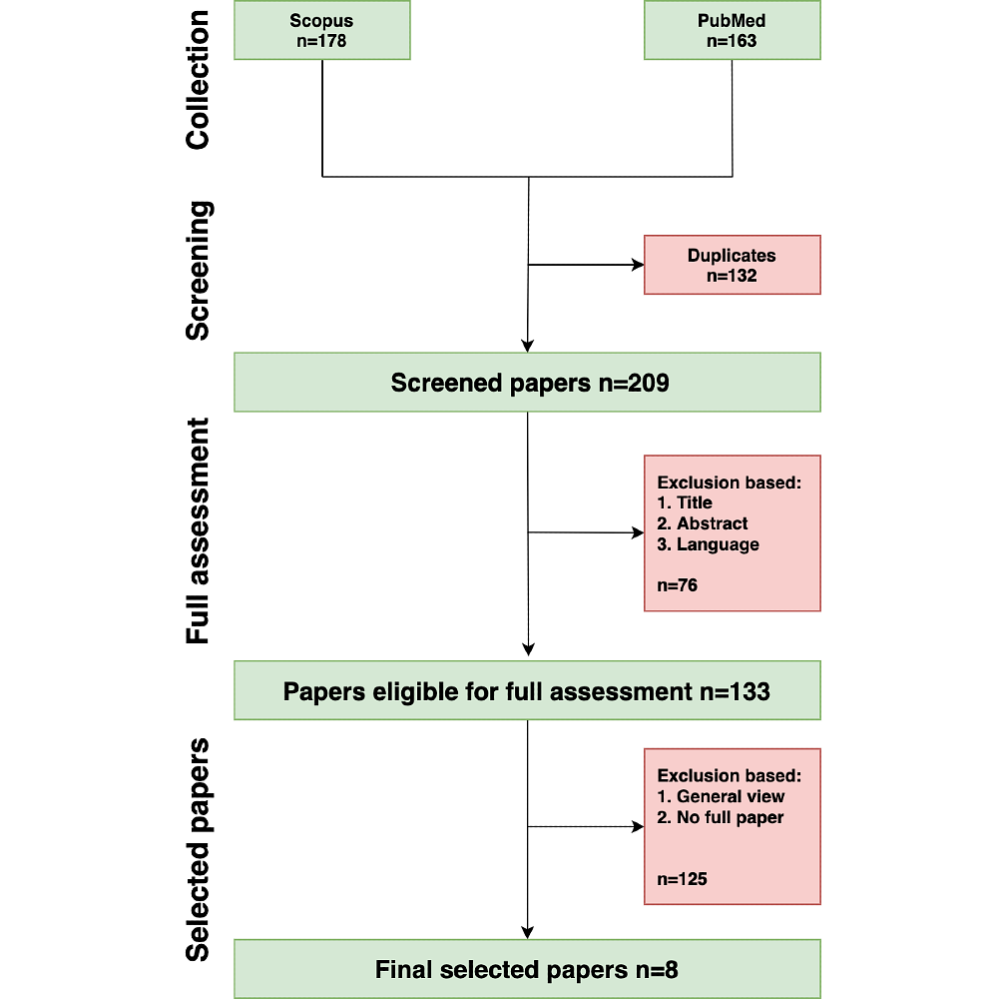
Process of scoping review.

We acknowledge that industries are also working on blockchain-based solutions within the health care sector, but often, the details of the frameworks are not disclosed. Therefore, in our research, we focus primarily on the academic sphere because the architecture frameworks and solutions are described in published work. Peer-reviewed articles, conferences, reviews and proceedings, and dissertations are included to provide a broad overview of different aspects of alert fatigue resulting from CDS. Only English papers were included, with no restrictions on the year or country of publication. We excluded general views, no full paper, and conference abstracts. The selection process for the scoping review is summarized in Figure 2.

## Results

### Related Work and Research Focus

Alert fatigue is a major problem faced by clinicians and is now a rising concern in the health care sector. The published literature on alert fatigue in the academic sphere started as early as 2007. We collected a total of 341 published items and finally selected a total of 8 [13,15,29-34] that fit our research criteria based on the scoping review in Figure 2. We then entered these items in Microsoft Word and Excel for deeper analysis. We summarize and sort these literatures according to their different key foci, methods, and benefits in Multimedia Appendix 2 [13,15,29-34].

Carli et al [35], Powers et al [36], and Hussain et al [37] pointed out that the high degree of alerts with low clinical relevance is one of the root causes of alert fatigue in their systematic literature reviews. This is because hospitals and other private health care institutions use or purchase commercial CDS tools to improve the overall quality of their health care systems. It is common for vendors and designers of commercial CDS tools to sharply restrict the ability to modify the setup for alert systems, resulting in a high volume of low-relevance alerts [2]. The strict, low-specificity settings imposed by vendors are due to their fear of being exposed to potential litigation if the removal of alerts fails to prevent a potential medication error.

One common attempt to address alert fatigue is to reduce the number of alerts of low clinical relevance by clustering alerts with similar clinical management options [32] or better specifications to generate useful alerts [31]. The machine learning algorithm–based CDS is another suggested method to generate more context-driven alerts [15] and patient-centric alerts [34]. Soundararajan et al [30] designed a blockchain architecture framework to leverage blockchain and smart contracts in support of clinical support tools that generate more patient context–appropriate alerts and thus generate fewer inappropriate alerts, which could reduce physician burnout. However, the actual benefits to patients and the extent of the positive impact on alert fatigue remain unclear.

All these efforts have managed to reduce the total number of alerts generated, but the fundamental issue of alert fatigue has still not been tackled. Bryant et al [38] pointed out that despite intensive efforts to reduce irrelevant alerts of commercial systems, overriding rates remain as high as reported over a decade ago. Medical experts suggested that improving alert fatigue should go beyond just reducing the total number of alerts [39].

Getting someone to attend the alerts is one way to reduce alert fatigue. Smithburger et al [5] suggested a potential strategy for directing alerts to medical professionals other than clinicians, for example, nurses. A study conducted in three academic medical centers in the Netherlands evaluated shifting time-dependent drug interaction alerts to medical staff such as nurses or pharmacists [40]. These results demonstrated the ability to improve the efficiency and effectiveness of such alerts and showed that incorrect administration times were reduced by 29% when they were directed at nurses. This can enable more collaborative treatment and decision care, whereas blockchain technology can be leveraged to enable alert sharing [25].

In our work, we have explored how blockchain can be leveraged to reduce alert fatigue by directing low-level alerts to patients in achieving high-quality collaborative clinical decisions. There has been a recent shift toward a more patient-centric data sharing for better collaborative decision making within the health care sector [41]. However, the relevant work remains limited. Thus, we contribute by designing an exploratory blockchain-based framework that enables low-level alert sharing with patients to enable more collaborative decision making while maintaining a high level of privacy and security. To design a sound framework, we need to understand and consider the challenges involved in facilitating the sharing of clinical alerts.

Data integrity and user privacy are two of the main concerns of the health care industry worldwide [42]. One of the reasons for this is that most of the current health care systems have weak and vulnerable centralized data storage procedures for preserving and managing sensitive medical data [43]. In 2019, the database of the Health Sciences Authority in Singapore was hacked for the third time in less than a year because of security loopholes, and more than 800,000 personal details were exposed [44]. Identity theft is another issue of concern in the health sector. According to Pandey et al [43], 10% of data breaches in the health industry in the last 10 years were categorized as identity theft.

There is a range of literature on blockchain-based frameworks that serves as an alternative to current vulnerable centralized database systems. EMRshare [45], Medchain [46], FHIRchain [25], and MedBlock [47] are examples of blockchain-based solutions that ensure high levels of data integrity and privacy for sharing medical records. In addition, smart contracts can enable a new service for health care to facilitate information sharing without a third party. For example, Medchain enables medical record access between multiple roles, such as patients, requesters, and health care providers, and helps them to achieve higher levels of efficiency and to satisfy security requirements [46]. This can improve collaborative decision making between different stakeholders, for example, clinicians and patients, in the health care sector.

Five key challenges must be addressed to develop a secure and effective blockchain framework and thus reduce the alert burden within health care. These 5 key challenges are as follows:

1. Data integrity
2. Privacy issues
3. Verifying and authenticating participants’ identities
4. Lack of secure information sharing
5. The extent of patients’ knowledge in the medical field

### Architectural Decisions to Address These Five Key Challenges

**Figure 3:**
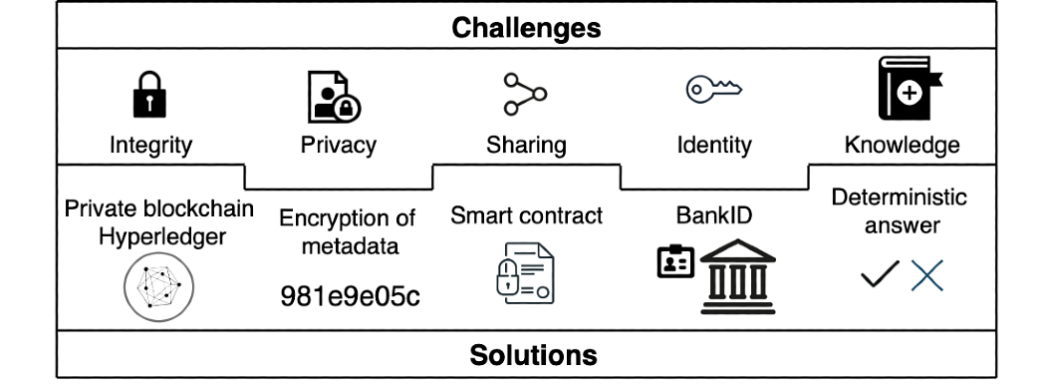
Architectural decisions in addressing the 5 key challenges.

In this section, we address the challenges suggested in the previous section. The architectural decisions are summarized in Figure 3.

#### Challenge 1: Data Integrity

##### Context

Health information is sensitive and must be highly secured, without the possibility of any data manipulation. Any alteration in a patient’s medical history could result in severe medication errors and even death. Medical data are often stored in and managed by centralized trusted third-party databases. However, such a centralized database can be vulnerable to single-point failures, resulting in loss or corrupted medical data and blocking of access deliberately during disputes by service providers [48]. Some modern EHR systems can be configured to have a backup and data redundancy mechanism that improves data storage resilience, but it requires additional configuration and maintenance that might be error prone due to human factors. According to some security experts, current systems in protecting our health data do not achieve the desired modern security standards [49].

##### Solution

A solution that offers resiliency out of the box and a tamper-sensitive storing environment to prevent any *silent* manipulation by making alterations obvious to members in the network.

##### Technical Requirement

Blockchain technology is a distributed ledger that contains replicated and synchronized digital data. It provides a platform for real-time data sharing between a large number of members in a network with a higher level of data trust [50,51]. *Data trust* denotes the reliability of the information and data provided [52]. A high level of data trust is important for decision making.

The data storage structure is a salient feature of blockchain, which ensures that information and data are stored in a tamper-evident environment [53]. All valid transactions are recorded in a block format, and each block is linked with a time stamp and hash references forming a chain of blocks [54]. Any attempt to alter information, for example, in the off-chain database, regardless of the intention, breaks the hash reference and thus makes it obvious to the other members of the network. This way, a hash reference creates a tamper-evident environment that maintains and ensures data integrity. The transactions recorded on the blockchain remain immutable and tamper-proofed owing to the structure and writing rights of the blockchain itself, which can guarantee a high level of data integrity.

#### Challenge 2: Privacy Issues

##### Context

Medical information, including medical records, prescription histories, patients’ personal information, and surgical records that are stored in digital formats, are classified as digital assets. This information requires high levels of privacy protection because it relates to the patient’s current physical or mental health and can reveal information about his or her health status [55]. Ensuring that current or new health services are in compliance with standards, such as General Data Protection Regulation (GDPR) is crucial to avoid unlawful behavior. For example, encryption, pseudonymization, or anonymization of personal data, whenever possible, to prevent unlawful data processing [55].

##### Solution

A private permissioned blockchain is a better option when it comes to ensuring on-chain data privacy and compliance with privacy regulations because transactions are visible only to members. Certain members of the network are granted permissions to read and write on the blockchain. By storing only metadata instead of actual health data, we can avoid exposing actual sensitive personal data, such as full name, diagnoses, and prescribed drugs, which could violate a patient’s privacy.

##### Technical Requirement

To increase the level of privacy protection, private blockchains such as Hyperledger are preferred over public blockchains, primarily because of the lower degree of visibility and level of *openness*. Information on private blockchains is only accessible to authorized members of the network and not just anyone with internet access. Only an authorized member, in our case clinician, has permission to write and store on the blockchain. This allows the framework to be more compliant with data protection regulations such as GDPR or HIPAA (Health Insurance Portability and Accountability Act) without compromising the privacy of patients [49].

Encrypting metadata in blockchain provides a higher level of security and protection for patients [25] because metadata are treated as sensitive data in health care. This prevents any unauthorized hacker from obtaining actual health information improperly. Encrypted metadata can act as a reference pointer to the patient’s prescription profile in the health system. The reference pointer links transactional data from the blockchain to the actual data stored on an off-chain database. This acts as a form of protection because it isolates the patients’ actual medical information from the reference itself. The pointer breaks and becomes invalidated when any alteration to the patient’s data occurs in the off-chain database. Another benefit in storing encrypted metadata is the lightweight reference pointer, which is more suitable and efficient to store on blockchain, which currently has limited storage capability. This can be a scalable alternative [25].

#### Challenge 3: Verifying and Authenticating Participants’ Identities

##### Context

It is important to ensure that the right patient receives the designated clinical alert from the clinicians. Clinicians working in hospitals can verify and authenticate themselves with the credentials offered by health care institutions through logging into the health care system. However, health care systems today lack a standard platform [56], particularly for patients, to verify and authenticate their digital identities.

##### Solution

Use a trusted digital identity management system to verify patients’ digital identities. Digital identity denotes the digital representation of entity attributes such as birth or other registered name, national ID number, and registered mobile number to access systems and applications using an identity mediation process [57-59]. This allows patients to authenticate their identities accurately and thus either authorize or revoke access to certain requestors. This is a way of protecting patients’ sensitive data, including managing their medical records, and it guarantees that security and privacy are compliant with local legislation and laws [60,61].

##### Technical Requirement

Federated digital identity management, registered once and trusted by many concepts, is widely used in consumer spaces such as Facebook and Google and is trusted by many applications [62]. Unlike traditional centralized identity management, users do not need to set up and register their digital identities with every service provider. In this system, mutual trust is established by receiving components of proof distributed by two or more centralized owners or by mutually recognizing each other’s trust and proofing standards [62]. Consortiums of leading banks and mobile operators have created private federated identification procedures, such as BankID in Norway [63] and Smart-ID in Estonia [64], to facilitate the distribution of verified and authenticated identities, thus enabling their citizens or users to access various portals, services, and platforms directly.

#### Challenge 4: Lack of Secure Information Sharing

##### Context

Each medical institution has its own way of governing medical records and data. Often, moreover, they are not interaccessible, thus making information sharing difficult. Along with strict legal regulations and the lack of trust in medical institutions outside the organization, information exchange becomes more challenging [45].

##### Solution

Use a common layer to enable information sharing securely without altering the current health care IT infrastructure and to enhance collaborative decision making.

##### Technical Requirement

Smart contracts can govern and facilitate information exchange between two different actors accurately and verifiably without the intervention of an intermediate third party. It also enables autonomous self-execution, once a set of predefined rules is met [65]. For example, when an alert is generated from CDS, it triggers a smart contract to direct the alert to the identified patient. The integration of smart contracts can increase the efficiency of members’ real-time decision making and overall information exchange. All events are recorded in the blockchain with a time stamp, and the blockchain structure can act as a common layer of information storage without changing the existing IT infrastructure. Smart contracts can track real-time performance and also query past events for the purposes of analysis.

#### Challenge 5: The Extent of Patients’ Knowledge in the Medical Field

##### Context

When directing alerts to patients for a collaborative decision, the main problem is that they may not have sufficient knowledge to make the correct decision. Making a wrong decision can be fatal to patients.

##### Solution

Only low-level and nonlife-threatening alerts are directed to patients governed by smart contracts. Patients will receive clinical alerts and then provide information back to the clinician. The aim of directing alerts to patients is bring the alert to their attention, instead risking its rapid dismissal by clinicians due to the high volumes of alerts. This could reduce alert fatigue and the total number of alerts because clinicians can place the emphasis on higher-level alerts.

##### Technical Requirement

Smart contracts execute actions by sending notifications to patients when the CDS generates an alert. The alert is then directed to the patient in the form of a question with a deterministic answer, either *Yes* or *No*. Given a real-time response, the clinician is able to modify the prescription accordingly and eliminate medication prescription errors based on the responses provided by patients.

### Principal Finding: MedAlert

#### Overview

This section provides an overview of MedAlert as a potential solution for reducing alert fatigue and enabling a more collaborative process of clinical decision making. This case study is developed as a two-step scenario: (1) *how a patient logs in with BankID to verify and authenticate his or her identity before revealing the alert* and (2) *how a patient is involved in the decision-making process.*

**Figure 4:**
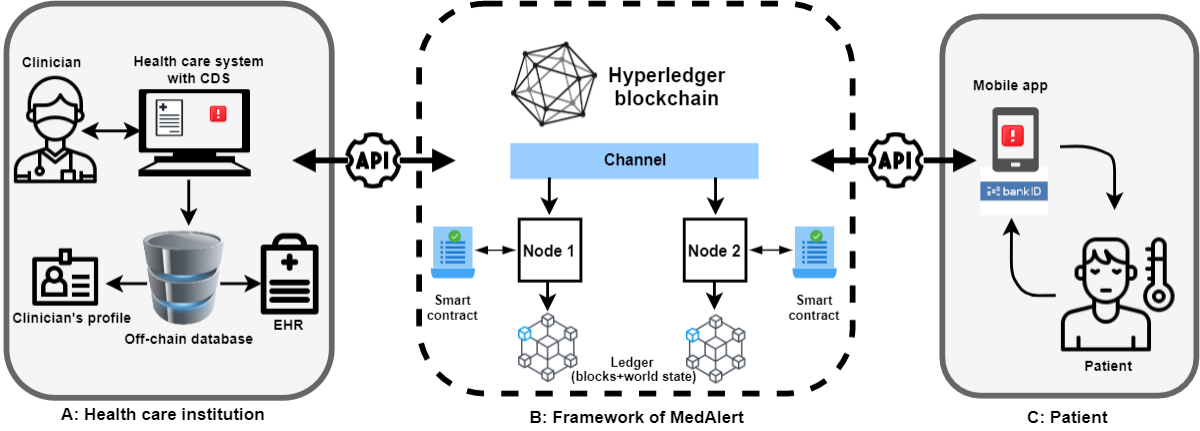
Overview of MedAlert. API: application programming interface; CDS: clinical decision support; EHR: electronic health record.

Figure 4 shows how MedAlert (B) enables the interaction between a clinician in a health care institution (A) and a patient (C). The MedAlert is hosted in a private blockchain framework such as Hyperledger. The clinician authorizes through logging into his or her profile with their credentials issued by the health care institution, whereas the patient can log in with BankID to verify and authenticate himself or herself. The blockchain nodes can be administered by a collection of health care organizations such as hospitals but not on a patient’s mobile device due the high requirement of computational resources and a consistent network connectivity. These nodes host ledgers and smart contracts that can be queried and updated by peer-connected applications.

Application programming interfaces (APIs) can enable alert sharing with multiple health care systems. Representational state transfer (REST) APIs can establish communication between mobile client apps and the blockchain network. A client app sends a transaction proposal using organization-specific REST APIs that enable apps to connect to nodes; invoke smart contracts that generate transactions; submit transactions to the network that will be ordered, validated, and committed to the distributed ledger; and receive events when this process is complete.

The consensus protocol in the private blockchain enables transaction data integrity. For every transaction, each node will verify that the transaction has been endorsed by the required organizations according to the endorsement policy of the smart contract that generated the transaction. For example, some transactions may only need to be endorsed by a single organization, whereas others may require multiple endorsements before they are considered valid. This process of validation verifies that all relevant organizations have generated the same outcome or result.

#### How a Patient Logs In With BankID to Verify and Authenticate His or Her Identity Before Revealing the Alert

**Figure 5:**
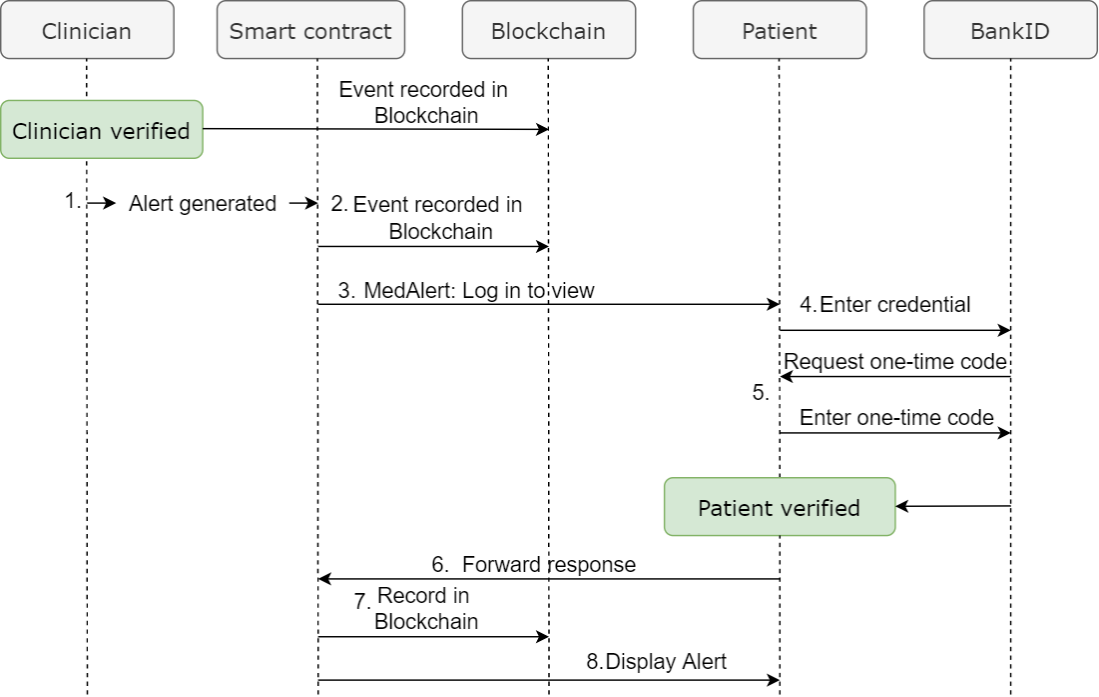
Workflow using BankID to verify and authenticate.

**Figure 6:**
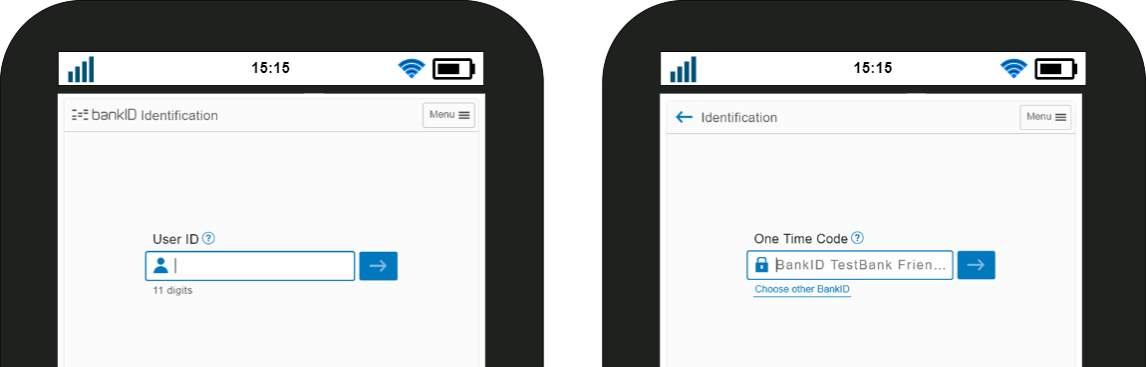
Personal credentials to verify identity (left) and request of one-time code to authenticate (right).

This section describes the step-by-step workflow, as shown in Figure 5. Before clinicians can access patients’ EHRs or prescribe new drugs, they need to authenticate their identities by logging in their credentials into the health care system. This event is recorded in the blockchain. When the clinician prescribes a drug to a patient and assumes that it could pose a threat to the patient:

1. A clinical alert (red exclamation mark) is generated from the CDS system, as shown in Figure 4. This triggers the smart contract.
2. This event is then recorded in the blockchain.
3. The smart contract also sends the alert to the patient’s registered mobile number.
4. The patient receives a message with a link to verify and authenticate his or her identity. Then he or she must log in to verify and authentic himself or herself by providing his or her registered user ID (eg, the 11 digits of a social security number) as sketched in Figure 6 (left).
5. The patient is then required to enter his or her one-time code for final authentication, as shown in Figure 6 (right).
6. When the authentication and verification is successful, the response is forwarded to the smart contract.
7. This event is also recorded in the blockchain.
8. The patient is then able to view the alert.

#### How a Patient Is Involved in the Decision-Making Process

**Figure 7:**
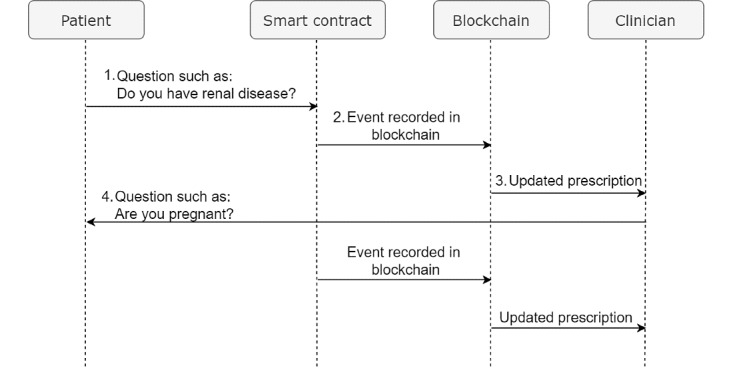
Workflow for involving a patient in the decision-making process.

After the patient has verified and authenticated his or her identity, the patient can access and read the information in the alert. The workflow is shown in Figure 7.

**Figure 8:**
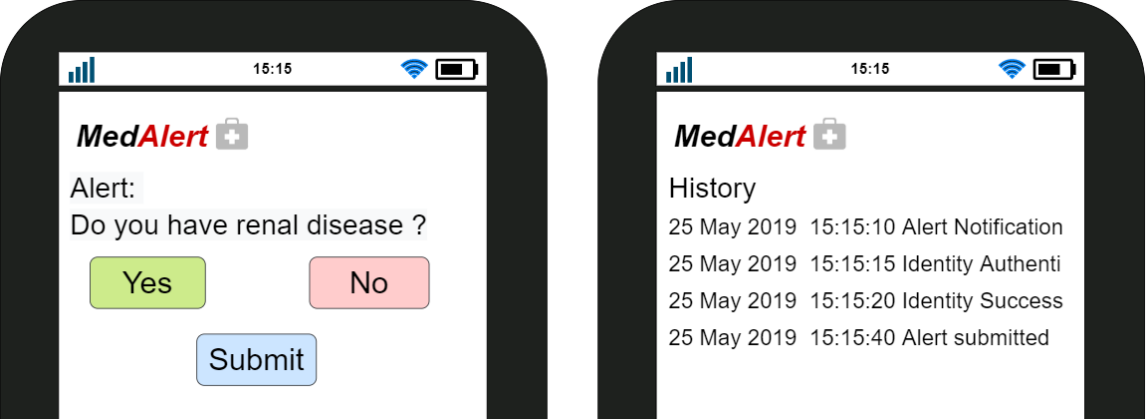
Question in alert (left) and history log of patients (right).

1. The first alert asks: “Do you have renal disease?” The answer to the question is either *Yes* or *No*, as shown in Figure 8 (left).
2. When the patient responds, the smart contract is then triggered, and the patient sends the response back to the clinician. The transaction is recorded in the blockchain.
3. The clinician updates the prescription according to the answer provided.
4. If another low-level alert pops up, the patient has to respond in real time before the prescription is finalized. The patient can view his or her history, as shown in Figure 8 (right).

## Discussion

### Comparison With Prior Work

Three different frameworks (MedAlert, MedRec, and MedAware) are compared in Table 1. These solutions can reduce the total number of alerts generated but with fundamentally different technologies. Both MedRec and MedAware focus on reducing alert fatigue by filtering irrelevant alerts. MedRec utilizes a smart contract embedded in a blockchain platform, from which CDS retrieves medical records via MedRec to retrieve relevant patient information and generate alerts that are more context based. MedAware uses a machine learning algorithm to flag more relevant and accurate alerts in real time after analyzing patients’ historical medical records. However, improving alert fatigue should go beyond just reducing the total number of alerts [39]. These two solutions only capture the clinician’s attention.

**Table 1:** Comparison of different framework solutions.

Solution		MedAlert	MedRec	MedAware
Alert reduction		Yes	Yes	Yes
**Alert capturing**				
	Clinician	Yes	Yes	Yes
	Patient	Yes	No	No
**Privacy**				
	Ownership	Clinician and patient	Clinician and patient	N/A^a^
	Encryption	Yes	No	N/A
**Blockchain**				
	Type	Private: Hyperledger	Public: Ethereum	No, machine learning
	Smart contract	Yes	Yes	N/A
	Miners	No	Yes, medical background	N/A

^a^N/A: not applicable.

Unlike MedRec and MedAware, MedAlert reduces alert fatigue by capturing the attention of both clinicians and patients. We believe that the way to reduce alert fatigue is to get the clinician’s attention, but there is no *perfect* solution in which clinicians are able to pay attention to all alerts [15], not even after the removal of irrelevant alerts. Therefore, MedAlert directs low-level alerts to patients and induces them to pay attention to provide real-time responses. This is a novel initiative moving toward a clinician-patient collaborative decision-making process to avoid potential medication errors resulting from action being overridden. This can improve the quality of the health care domain with respect to better patient outcomes and reducing physician burnout.

MedAlert runs on the private Hyperledger blockchain, which ensures a higher privacy compared with MedRec, which runs on the public Ethereum blockchain. This is because private blockchain is better suited to a highly regulated industry such as health care due to the stricter requirements regarding patient privacy and data protection. To avoid information leakage, both MedAlert and MedRec record only metadata or reference pointers rather than patient’s medical data on blockchain. To enhance patients’ data privacy, all metadata is encrypted and stored on MedAlert blockchain, where only authenticated patients can view the transactions and authorized clinicians can read and write transactions. This makes MedAlert better compliant to standards such as GDPR (Art. 32. Security of processing) compared with MedRec.

Apart from ensuring a higher-level privacy environment, MedAlert, deployed in private Hyperledger, has a better performance than MedRec, deployed in Ethereum. The assessments from Pongnumkul et al [66] show that Hyperledger outperforms Ethereum in 3 evaluation metrics: execution time, latency, and throughput. For example, the average latency of Ethereum is about 2 times at a low number of transactions and can increase up to 14 times that of Hyperledger at a high number of transactions. This is important when fast information sharing is needed between a clinician and a patient during collaborative decision making.

MedAlert can improve the flow of communication between clinicians and patients. Clinicians may need to ask for and validate information with patients because without this step, there is a significant risk of error in ordering or prescribing medication [67]. This risk can increase when alerts generated by CDS are simply overridden. MedAlert can reduce this and prevent it from happening by sharing clinical alerts with patients. Patients can receive the alert and be asked to provide information. If they are uncertain, they can enter into direct communication with the clinician and deal with the alert that way.

### Future Work

Validation work such as threat analysis is needed in future work to elucidate the effectiveness and the potential vulnerabilities of using MedAlert before deploying it in the eHealth sector [68]. This would provide a documented performance evaluation of MedAlert to persuade health care leaders of the benefits of this new digital tool and gain sufficient support from them for its deployment. Despite numerous published literature on how blockchain can record immutable transactions and enhance interoperability and thus improve health care, many leaders remain unsure about what blockchain has to do with health care. Proof of validation is an important step in scaling up this framework and making it applicable to the real world [69].

Second, sorting and tiering alerts based on severity, for example, sorting into 3 tiers: low, mid, and high, are needed as a part of future work to validate MedAlert. This is to determine which low-level alerts are suitable for patients because clinicians tend to accept high-severity alerts slightly more often than mid- or low-severity interaction alerts [11]. However, the process of tiering alerts is highly subjective when it comes to deciding which alerts are considered low level and time consuming for all medical experts before reaching a common consensus. Thus, this initial step in selecting which alerts are to be shared with patients can be challenging.

Decentralized identity management is an alternative way of verifying and authenticating users. It eliminates the limitations of centralized identity systems, helps achieve compliance with the most comprehensive national data protection laws, and returns ownership and control of identity data back to the individual. Various decentralized identity management systems exist that provide solutions using a distributed ledger technology. Evernym [70], uPort [71], and Sovrin [70] are some examples of identity projects that are working on decentralized identity platforms. However, these sophisticated solutions are still at a provisional stage, where more validation, discussion, and investigation are needed [60].

### Limitations

MedAlert is suitable for a specific group of users. Collaborative decision making may be challenging for patients who are less technology savvy, particularly for elderly patients, who may not be able to use MedAlert effectively. For example, the steps where patients need to verify and authenticate themselves and thus gain access to alerts could be confusing for the elderly and may induce unnecessary stress on them. MedAlert is not suitable for in-patients either where they require constant monitoring. This is because they may not be able to provide a response when they are unwell in the hospital.

Directing low-level alerts to patients may create ethical issues where the responsibility is indirectly shifted on to them in cases when they provide incorrect responses. In a study conducted in medical centers in the Netherlands where alerts were directed to nurses, despite improvements in efficiency and effectiveness, the study concluded that such alerts should not be directed to nurses [40]. It is difficult to find the right balance of responsibilities between clinicians, nurses, and patients in a collaborative decision-making process.

Privacy concerns are covered by the GDPR. Storing digital assets, such as medical records on blockchain, could violate personal privacy. Although MedAlert only stores patients’ metadata on blockchain, it is not entirely anonymous. Malicious acts include attempting to learn about and identify actual personal patients based on the pseudo-anonymous information on blockchain. In addition, the permanent storage of information, both data and metadata, belonging to a person could violate GDPR (Art. 17 Right to erasure or to be forgotten) in cases when users want to have their data completely erased or deleted.

### Conclusions

CDS supports the decision-making process in preventing medication errors by generating alerts. Clinicians can now rely on these alerts along with their knowledge and past experience to avoid medication errors. Due to the low specificity and highly restricted modifications of the CDS setting, a high volume of irrelevant alerts has caused clinicians to experience alert fatigue. This results in a high overriding rate, which can cause medication errors.

From our scoping review, we found different methods of reducing the number of alerts, such as machine learning algorithms and blockchain technology, by filtering out irrelevant alerts. We developed a different solution that is similar to what medical experts pointed out, where improving alert fatigue should go beyond just reducing the total number of alerts.

In line with this idea, we designed MedAlert, a blockchain-based solution, by sharing low-level alerts with patients where clinicians typically have a greater tendency to override low-level alerts. The goal is to ensure that alerts catch the attention of both patients and clinicians, thus preventing medication errors, instead of being habitually overridden. In our own work, we introduced a second layer by engaging patients in providing a response and making them, at least, partially responsible for alert verification. This second layer reduces alert fatigue of clinicians and, at the same time, engages patients in the collaborative process, making it harder for medication errors to occur.

Other potential advantages of MedAlert over other frameworks include ensuring a greater degree of patient privacy and the ability to establish a new communication layer between patients and clinicians. Smart contracts and the use of BankID (federated identity management) are useful in authenticating patients and ensuring that the right person receives the alert.

Directing alerts to patients faces challenges such as finding a balance between patients and clinicians without raising ethical issues. This solution may not be suitable for elderly patients or in-patients where they require constant monitoring. Sorting and tiering the alerts based on levels of severity is also challenging because it is subjective and may vary between different panels of medical experts.

For the health care sector to benefit from the potential value of this innovative idea, future work, for example, on the validation of MedAlert based on real-world scenarios, such as the degree of compliance with GPDR, is needed. Providing documented evaluations of the performance of MedAlert is crucial to gain the support of health care leaders in nurturing this idea as a potential solution to reducing alert fatigue.

## Appendix

Multimedia Appendix 1 Search strings.

Multimedia Appendix 2 Summary of selected literature.

## Abbreviations

API: application programming interface
CDS: clinical decision support
EHR: electronic health record
GDPR: General Data Protection Regulation
IT: information technology
NTNU: Norwegian University of Science and Technology
REST: representational state transfer
