# Supplementary material for: Reducing Alert Fatigue by Sharing Low-Level Alerts With Patients and Enhancing Collaborative Decision Making Using Blockchain Technology: Scoping Review and Proposed Framework (MedAlert)
Source: J Med Internet Res. 2020 Oct 28;22(10):e22013. doi: 10.2196/22013 (PMC7657729; doi:10.2196/22013)
Supplement: Multimedia Appendix 1 [file jmir_v22i10e22013_app1.doc]

**Multimedia Appendix 1.** Search strings.

**Key words:**

1. Blockchain

2. Clinical decision support

3. Alert burden

4. Alert fatigue

| ***Database*** | **Search string** | **Hits** |
| --- | --- | --- |
| **Scopus**  **Search box :**  **Article title, Abstract, Keywords:** | blockchain AND “clinical decision support” | 5 |
|  | blockchain AND “alert burden” | 0 |
|  | blockchain AND “alert fatigue” | 1 |
|  | Alert burden AND “clinical decision support” | 20 |
|  | Alert fatigue AND “clinical decision support” | 151 |
|  | blockchain AND “alert burden” AND “clinical decision support” | 0 |
|  | blockchain AND “alert fatigue” AND “clinical decision support” | 1 |
| **PubMed**  **Search Box:**  **All fields** | blockchain AND “clinical decision support” | 1 |
|  | blockchain AND “alert burden” | 0 |
|  | blockchain AND “alert fatigue” | 0 |
|  | Alert burden AND “clinical decision support” | 20 |
|  | Alert fatigue AND “clinical decision support” | 142 |
|  | blockchain AND “alert burden” AND “clinical decision support” | 0 |
|  | blockchain AND “alert fatigue” AND “clinical decision support” | 0 |
