# Supplementary material for: Reducing Alert Fatigue by Sharing Low-Level Alerts With Patients and Enhancing Collaborative Decision Making Using Blockchain Technology: Scoping Review and Proposed Framework (MedAlert)
Source: J Med Internet Res. 2020 Oct 28;22(10):e22013. doi: 10.2196/22013 (PMC7657729; doi:10.2196/22013)
Supplement: Multimedia Appendix 2 [file jmir_v22i10e22013_app2.docx]

| **Authors** | **Year** | **Key Focus** | **Method** | **Solution** | **Benefits** | **Comments** |
| --- | --- | --- | --- | --- | --- | --- |
| Chen, Chokshi, Hegde, Gonzalez, Iturrate, Aphinyanaphongs and Mann [29] | 2020 | To increase the signal-to-noise ratio of CDS alerts | EHR–integrated  machine learning algorithm | SmartCDS  System : suppresses low-value vaccination alerts | Suppressed 43.67%  potential vaccination alerts without detriment to overall order rates. | Lacks the explanation on the selection of shingles vaccination alerts as a choice of study. |
| Chaparro, Hussain, Lee, Hehmeyer, Nguyen and Hoffman [13] | 2020 | Reducing interruptive alert burden | Quality improvement | Interactive dashboard to monitor alert frequency | Reduced 25 unique alerts by more than 35% | Lacks evaluation on patient care outcomes after reducing alerts |
| Segal, Segev, Brom, Lifshitz, Wasserstrum and Zimlichman [15] | 2019 | Reducing drug prescription error and adverse drug events | Machine-learning algorithm-based CDS | MedAware, commercial software | Generates high alert accuracy (up to 85% of alerts are clinically valid) | Only conducted in one department in a hospital. Deploying in another department for comparative analysis as future work |
| Soundararajan, McDaniel, Shin, Sneha and Soundararajan [30] | 2019 | Improve clinical decision support system | Literature review and architectural framework | MedRec (smart contract on Ethereum blockchain and API library) | Produce context-driven alerts, fewer inappropriate alerts | Lacks the implementation and validation of this proof of concept. Security of the patient data issue is highlighted in their work |
| Heringa, van der Heide, Floor-Schreudering, De Smet and Bouvy [31] | 2018 | To reduce the number of alerts with better specification | Two-step consensus process by a panel of experts to determine if a new alert is needed for (re)assessments | Alert CDS simulation | 93% lower alert rate compared with the original CDSS for the ten concerning drug interaction | Different expert group could reach different consensus regarding which event requires alerts and which does not. Simulation study is different from real world scenario |
| Heringa, Siderius, Floor-Schreudering, M de Smet and Bouvy [32] | 2017 | Lower alert generated by CDS | Retrospective analysis: clustering drug interaction alerts with similar management recommendations | Alert CDS simulation | Overall alert rate dropped by 10%, corresponding to a decrease of 21 alerts per day. | Reducing alerts through clustering without assessing the clinical relevance of the alerts |
| Khalifa and Zabani [33] | 2016 | Improving CDS by reducing alert fatigue | Literature review and qualitative survey: to collect opinions, experience and suggestions | Electronic website portal and semi-structured interviews | Classifying alerts based on severity, passive or active.  Automated system and learning mechanism | Recommendation from experts. Lacks evaluation and validation work |
| Jafarpour, Abidi, Ahmad and Abidi [34] | 2015 | Avoid alert fatigue by managing alerts from one or more CDS | Framework: INITIATE (Intelligent Adaptive Alert Environment) | Machine-learning algorithm, web ontology language coupled with alert management engine | Filter out alerts that are too old. Aggregates alerts from different CDS | Lacks evaluation on patient safety outcomes after reducing alerts |

**Multimedia Appendix 2.** Summary of selected literature.
